# A single origin of the photosynthetic organelle in different *Paulinella *lineages

**DOI:** 10.1186/1471-2148-9-98

**Published:** 2009-05-13

**Authors:** Hwan Su Yoon, Takuro Nakayama, Adrian Reyes-Prieto, Robert A Andersen, Sung Min Boo, Ken-ichiro Ishida, Debashish Bhattacharya

**Affiliations:** 1 Bigelow Laboratory for Ocean Sciences, West Boothbay Harbor, Maine, USA; 2 Graduate School of Life and Environmental Sciences, University of Tsukuba, Tsukuba, Ibaraki, Japan; 3 Department of Biology and Roy J. Carver Center for Comparative Genomics, University of Iowa, Iowa City, Iowa, USA; 4 Department of Biology, Chungnam National University, Daejeon, Korea

## Abstract

**Background:**

Gaining the ability to photosynthesize was a key event in eukaryotic evolution because algae and plants form the base of the food chain on our planet. The eukaryotic machines of photosynthesis are plastids (e.g., chloroplast in plants) that evolved from cyanobacteria through primary endosymbiosis. Our knowledge of plastid evolution, however, remains limited because the primary endosymbiosis occurred more than a billion years ago. In this context, the thecate "green amoeba" *Paulinella chromatophora *is remarkable because it very recently (i.e., minimum age of ≈ 60 million years ago) acquired a photosynthetic organelle (termed a "chromatophore"; i.e., plastid) *via *an independent primary endosymbiosis involving a *Prochlorococcus *or *Synechococcus*-like cyanobacterium. All data regarding *P. chromatophora *stem from a single isolate from Germany (strain M0880/a). Here we brought into culture a novel photosynthetic *Paulinella *strain (FK01) and generated molecular sequence data from these cells and from four different cell samples, all isolated from freshwater habitats in Japan. Our study had two aims. The first was to compare and contrast cell ultrastructure of the M0880/a and FK01 strains using scanning electron microscopy. The second was to assess the phylogenetic diversity of photosynthetic *Paulinella *to test the hypothesis they share a vertically inherited plastid that originated in their common ancestor.

**Results:**

Comparative morphological analyses show that *Paulinella *FK01 cells are smaller than M0880/a and differ with respect to the number of scales per column. There are more distinctive, multiple fine pores on the external surface of FK01 than in M0880/a. Molecular phylogenetic analyses using multiple gene markers demonstrate these strains are genetically distinct and likely comprise separate species. The well-supported monophyly of the *Paulinella chromatophora *strains analyzed here using plastid-encoded 16S rRNA suggests strongly that they all share a common photosynthetic ancestor. The strain M0880/a is most closely related to Japanese isolates (Kanazawa-1, -2, and Kaga), whereas FK01 groups closely with a Kawaguchi isolate.

**Conclusion:**

Our results indicate that *Paulinella chromatophora *comprises at least two distinct evolutionary lineages and likely encompasses a broader taxonomic diversity than previously thought. The finding of a single plastid origin for both lineages shows these taxa to be valuable models for studying post-endosymbiotic cell and genome evolution.

## Background

The origin of the first photosynthetic organelle (plastid) is explained by primary endosymbiosis, whereby a non-photosynthetic protist engulfed and retained a cyanobacterium as a cytoplasmic organelle [1,2]. A variety of plastid-derived and some nuclear sequence data, as well as plastid-associated traits such as the composition and evolutionary history of the protein translocons [3], and solute transporters embedded in the inner organelle membrane suggest that descendants of this 'primary' photosynthetic eukaryote were the ancestors of the putative supergroup Plantae. The Plantae is comprised of the red, green (including land plants), and glaucophyte algae [4,5]. Via eukaryote-eukaryote (secondary and tertiary) endosymbiosis, photosynthesis and its associated genes spread thereafter into other eukaryotic groups (e.g., euglenoids, diatoms, and dinoflagellates; [6-10]). Despite its importance to eukaryote evolution, our understanding of primary plastid origin remains limited because the endosymbiosis occurred > 1 billion years ago [11,12]. This dilemma has, however, a potential solution given the recently clarified evolutionary history of *Paulinella chromatophora*. This little-known testate, filose amoeba [13], which is a cercomonad species (supergroup Rhizaria), contains two blue-green photosynthetic inclusions termed chromatophores [14]. There are no known fossils of *Paulinella*, however, euglyphid-like testate amoebae have a long fossil history, occurring in sediments dated from 742 – 770 million years ago [15,16]. Importantly, *P. chromatophora *is the only known case of an independent primary photosynthetic organelle acquisition (from a prey cell related to extant *Prochlorococcus*/*Synechococcus*-like cyanobacteria), putatively recapitulating the process that gave rise to the Plantae plastid [17-20]. This makes *P. chromatophora *an outstanding model for elucidating plastid acquisition and post-endosymbiotic genome evolution. Two key reasons why it is believed the chromatophores (= plastids) of *P. chromatophora *are *bona fide *organelles rather than temporary photosynthetic inclusions (for details, see [21]) are the apparent regulation of plastid division by the amoeba and the loss of 2/3 of plastid coding potential through outright gene loss or transfer to the nucleus (i.e., from ca. 3 Mb in free-living *Prochlorococcus*/*Synechococcus *cyanobacteria to 1.02 Mb in the *P. chromatophora *plastid, [22]). *Paulinella *plastid genome size is, however, far greater than the ca. 100–200 Kb for typical plastid genomes from algae and plants suggesting it is likely a "work in progress" (for details, see [23]).

Since its discovery by Lauterborn [14], four different *Paulinella *species have been reported – the photoautotrophic *P. chromatophora*, which contains two plastids, is clearly separated from its three heterotrophic sister species that lack a plastid (i.e., *P. ovalis*, *P. intermedia*, and *P. indentata*), although all share a typical oval-shaped cell morphology that consists of five rows of silicate scales (see Table 1, [24-26]). The phylogenetic relationship between these four *Paulinella *species is unknown because of a lack of sequence data from heterotrophic *Paulinella *species. However, it is obvious that *P. chromatophora *(as a representative of the genus *Paulinella*) and *Euglypha *are closely related based on nuclear SSU rDNA trees [13,27]. The derived position of *P. chromatophora *among the Paulinellidae and the known ability of *P. ovalis *to ingest cyanobacteria [25] make it a reasonable assumption that the primary plastid endosymbiosis occurred in *P. chromatophora *after its split from heterotrophic ancestors.

**Table 1:** Comparison of morphological characters among *Paulinella *species.

	This study	*P. chromatophora*		*P. ovalis*	*P. intermedia*	*P. indentata*
	FK01 (n = 17)	M0880/a (n = 13)	Lauterborn 1895, Kies 1974	Johnson et al 1988	Vørs 1993	Hannah et al 1996
Plastid	+	+	+	-	-	-

Length (μm)	15 – 17	23 – 27	20 – 30	4.5	6.6	11 – 17
Width (μm)	10 – 11	16 – 20	15 – 20	3	2.9	8 – 10
No. of scales/column	10 – 11	12 – 14	11 – 12	5 – 6	6 – 7	6 – 7
No. of columns	5	5	5	5	NA	Staggered rows
Oral scales	5	3	3	3	3	2 – 4
Fine-pored external scales	+ (distinct)	+ (not distinct)	NA	+	-	+ (3 – 4 rows of pores at the end of scales)
Scale feature	"Sieve-plate" on the internal surface	"Sieve-plate" on the internal surface	"Sieve-plate" on the internal surface	One-ridges and hollow scales	Smooth scales	Two-ridges and hollow scales

The minimum age of the endosymbiosis is postulated to be ca. 60 Ma based on the mode and tempo of plastid genome reduction [22]. Given these data, it is of high interest to isolate other photosynthetic *Paulinella *strains/species to facilitate in-depth study of post-endosymbiotic genome evolution in distinct lineages that potentially share a common ancestral endosymbiont. *Paulinella chromatophora *has been reported from around the world, including sites in Switzerland [28], the United Kingdom [29], and the United States [30,31], however these were simple statements of occurrence without the deposition of voucher samples. This depauperate history of collection apparently reflects the rarity of *P. chromatophora *in nature and difficulties in its culture. Therefore, all morphological and ultrastructural studies stem from samples collected in Germany [19,32]. Recent molecular phylogenetic and genomic studies also relied on strain M0880/a that was isolated in Germany [13,17,22,33,34]. Here, we isolated photosynthetic *Paulinella *cells from several freshwater sites in Japan and established a new strain in culture (FK01). We then conducted comparative morphological and molecular phylogenetic analysis of these taxa, focusing on the Japanese FK01 and the German M0880/a strains.

## Results and discussion

### Morphology and ultrastructure of Paulinella

**Figure 1:**
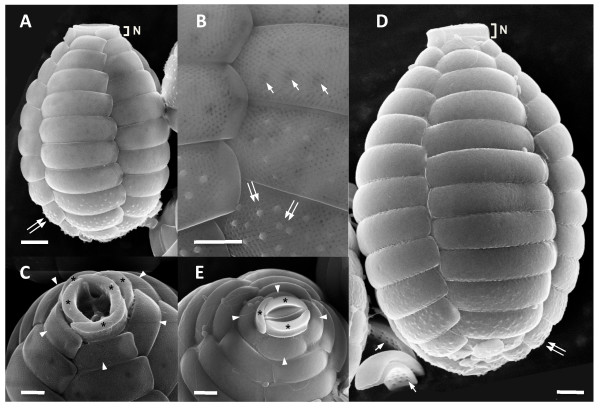
**SEM images of photosynthetic *Paulinella *strains FK01 (A – C) and M0880/a (D, E)**. FK01 is smaller in cell size than M0880/a (i.e., the scale bar is the same in A and D). Distinctive, multiple fine pores are present on the surface of scales of FK01 (B). The five (C) oral scales (asterisks) are shown from FK01, whereas only three (E) are found in M0880/a. The projecting oral scales (N), first row of body scales (arrow head), "sieve-plate" in internal surface (arrow), and pustules (double arrow) are indicated. Scale bars (A, D, E = 2 μm; B, C = 1 μm).

Morphological comparisons between *Paulinella *FK01 and M0880/a strains were done using scanning electron microscopy (SEM). The general morphological characters we observed for M0880/a (Fig. 1D, E) are similar to the original description [14] and a previous study [19]. The test is ovoid (23 – 27 × 16 – 20 μm; n = 13) and covered with silica scale plates. The anterior end has a narrow aperture (3 – 5.6 μm) that is comprised of three oral scales (see asterisks in Fig. 1D) extending from the cell body as a "neck" (see N in Fig. 1D). Within this neck, two slightly curved oral scales abut each other between a membranous operculum with the third scale covering the edge of the two oral scales (Fig. 1E). Below the oral scales, five descending columns of scales cover the test. A total of 12 – 14 scales are found in each column with the first row containing four scales instead of five (see arrow head in Fig. 1D). The scales around the anterior and posterior cell regions are smaller than in the middle. Three or four rows of scales from the posterior end have dozens of pustules on the surface of each scale. The external surface of the rest of the body scales is smooth. The internal surface of the scales that are shown in Fig. 1D (see arrows in inverted scales) have 12 – 18 pores per scale which were reported as "sieve-plates" in a previous study [19].

Cells of strain FK01 are clearly distinguished from the original description of *P. chromatophora *(i.e., M0880/a). These *Paulinella *cells are of a relatively smaller size (15 – 17 × 10 – 11 μm; n = 17) than in M0880/a (see, Fig. 1A–C) and there are between 10 – 11 scales per column. There are more distinctive, multiple fine pores on the external surface of scales in FK01 than in M0880/a (see, Fig. 1B). The fine-pored external surface was reported in newly formed scales in the heterotrophic species *P. ovalis *[25]. Another heterotrophic species, *P. indentata *has a single row of fine-pores along the scale but three or four rows on the end of the scales [24]. There are five oral scales in the aperture (2.4 – 3.6 μm), which is comprised of two main scales between a membranous operculum and three thinner scales that cover the main oral scales (see asterisks in Fig. 1C). The oral scales of FK01 are not projected outward to the same extent as in M0880/a (Fig. 1A vs. 1D, indicated with N), and the first row of body scales consists of five not four scales (see arrow head in Fig. 1A, C). Pustules on the four rows of posterior scales (see double arrow in Figs. 1A–B) are more distinct than those in the M0880/a strain. The size of pustule-covered scales gradually increases from the centre to the outside in a counter-clockwise direction. Around 10 – 20 internal "sieve-plate" pores are also found in FK01 and are detectable in SEM images taken from outside the cell, particularly in the posterior region (see arrows in Fig. 1B).

Table 1 shows a comparison of key morphological characters from different *Paulinella *species. Traits such as cell size, number of scales per column, and number of oral scales have been used to define species. For example, *P. intermedia *is similar to *P. ovalis *in size and the number of scales per column but it differs from the latter species by possessing flat scales and a wider oral aperture [26]. Due to the presence of two plastids in the cytosol, the FK01 and M0880/a strains are clearly distinguished from other *Paulinella *species. In turn, FK01 is distinct from M0880/a with regard to cell size, number of scales, number of oral scales, and by having distinct fine-pores in the body scales.

### Molecular phylogenetic analysis

**Figure 2:**
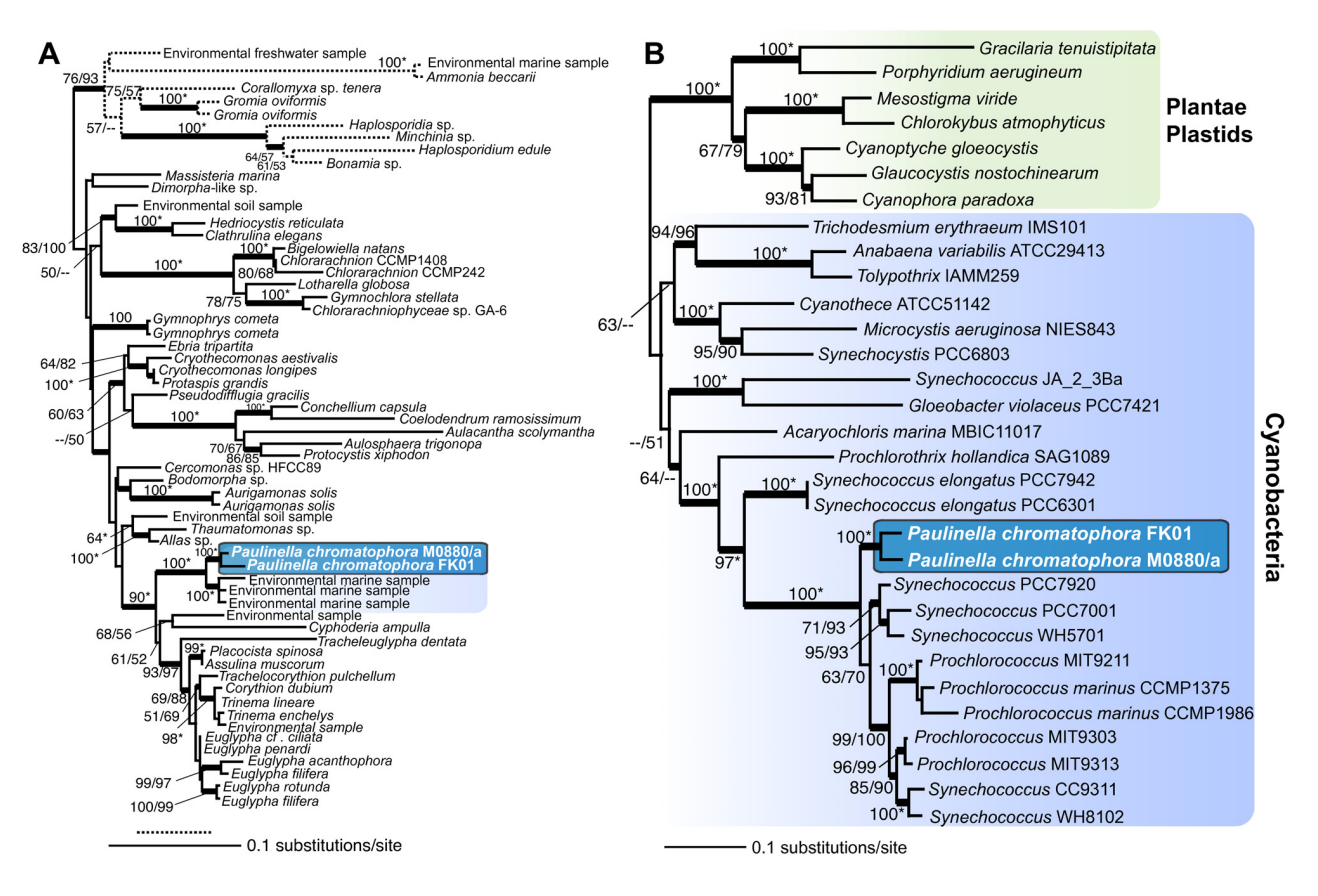
**(A) RAxML phylogenetic tree of nuclear 18S rDNA from Rhizaria with the root placed on the branch leading to the Foraminifera**. (B) RAxML phylogenetic tree of a concatenated data set of plastid-encoded 16S + 23S rDNA with the root placed on the branch leading to Plantae plastids. The numbers at the nodes of both trees show support values derived from a RAxML bootstrap analysis followed by those from a PhyML analysis. When both values are the same, then this is marked with an asterisk and when a node is not resolved with a method than this is denoted with dashes. Only bootstrap values ≥ 50% are shown. The thick branches have a Bayesian posterior probability > 0.95. Branch lengths are proportional to the number of substitutions per site (see scale bar).

Given the obvious morphological differences described above, we used gene sequences to test the evolutionary relationship between the two strains. Multiple markers were used for this purpose and we first present maximum likelihood (ML) trees inferred from nuclear 18S rDNA and a concatenated data set of plastid 16S + 23S rDNA (Figs. 2A, B). Given that filose amoebae such as *P. chromatophora *and *Euglypha *are consistently recovered as members of the Rhizaria (e.g., [13,27]), we chose to include only 18S rDNA sequences from members of this putative supergroup [35]. The 18S rDNA tree shows a monophyletic grouping of the two *Paulinella *strains, suggesting they share a common photosynthetic 'host' ancestor. This hypothesis is substantiated by the plastid rDNA tree (Fig. 2B), that shows a monophyletic grouping of M0880/a and FK01 as sister to *Synechococcus*- and *Prochlorococcus*-type cyanobacteria as previously described for M0880/a [22,34]. The 18S rDNA tree provides high bootstrap and Bayesian support (posterior probability > 0.95 for all thick nodes in the trees; 100% bootstrap support in both RAxML [RML] and PhyML [PML] analyses) for a clade that unites both *P. chromatophora *strains with a group of uncultured marine environmental samples (i.e., GenBank accession numbers; AB275059, EF526891, see [36]). Because there are no sequence data available from heterotrophic *Paulinella *species with a taxonomic identification, we could not provisionally identify the source of the environmental samples. However, given that all described photosynthetic *P. chromatophora *are derived from freshwater environments [14,19] and the present work], these environmental sequences likely represent a marine sister group within the Paulinellidae (e.g., *P. ovalis*, *P. indentata *[24,25]). The Paulinellidae is closely related (90% RML, PML) to other euglyphids such as *Tracheoeuglypha*, *Cyphoderia*, and *Euglypha *species.

**Figure 3:**
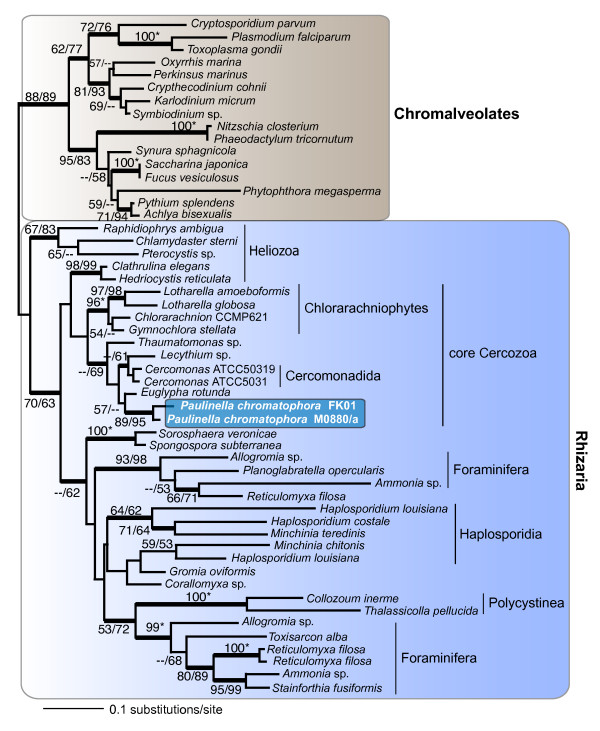
**RAxML phylogenetic tree of actin sequences from photosynthetic *Paulinella *spp. with the root placed on the branch leading to chromalveolate taxa (i.e., stramenopiles + alveolates)**. The numbers at the nodes show support values derived from a RAxML bootstrap analysis followed by those from a PhyML analysis. When both values are the same, then this is marked with an asterisk and when a node is not resolved with a method than this is denoted with dashes. Only bootstrap values ≥ 50% are shown. The thick branches have a Bayesian posterior probability > 0.95. Branch lengths are proportional to the number of substitutions per site (see scale bar).

**Figure 4:**
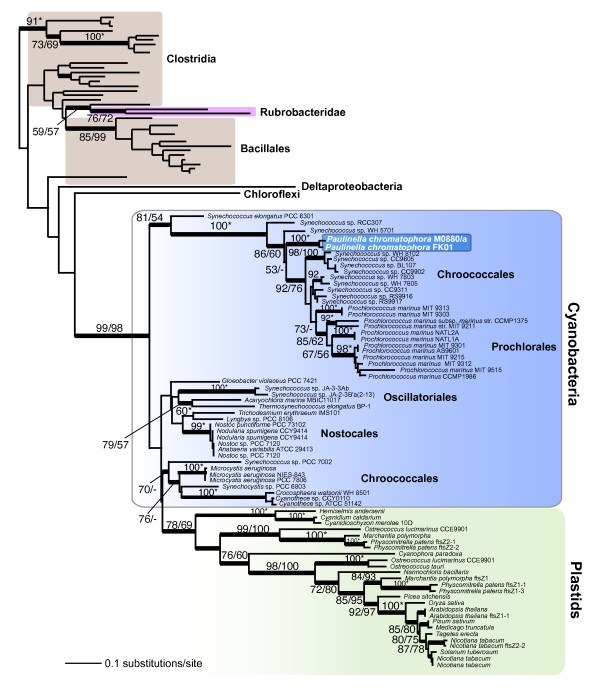
**RAxML phylogenetic tree of plastid-encoded *ftsZ *from photosynthetic *Paulinella *spp. with the root placed on the branch leading to Clostridia**. The numbers at the nodes show support values derived from a RAxML bootstrap analysis followed by those from a PhyML analysis. When both values are the same, then this is marked with an asterisk and when a node is not resolved with a method than this is denoted with dashes. Only bootstrap values ≥ 50% are shown. The thick branches have a Bayesian posterior probability > 0.95. Branch lengths are proportional to the number of substitutions per site (see scale bar).

Trees inferred from actin and *ftsZ *protein sequences are shown in Figures 3 and 4, respectively. The actin tree provides both bootstrap and Bayesian support for the existence of a branch that unites M0880/a and FK01 (89% RML, 95% PML), which in turn is sister to other euglyphid taxa (57% RML, absence of Bayesian support) within the Rhizaria. The overall topology for Rhizaria actins (Fig. 3) is consistent with previous analyses [37]. This monophyletic clade, although surrounded by many internal nodes that are only weakly supported, shows unequivocal sequence divergence between M0880/a and FK01. Pairwise analysis of synonymous (Ks) and non-synonymous (Ka) substitution rates between the *Paulinella *actin coding regions are 1.0023 and 0.0181, respectively (Ka/Ks = 0.0181). This ratio is comparable to actin sequence differences between two green algal *Ostreococcus *species (i.e., *O. tauri *vs. *O. lucimarinus*; Ks = 1.2489, Ka = 0.0085, Ka/Ks = 0.0068), two multicelluar liverworts (*Pellia endiviifolia *vs. *P. borealis*; Ks = 0.8396, Ka = 0.0037, Ka/Ks = 0.0044), and different genera of yeasts (*Saccharomyces *vs. *Kluyveromyces*, Ks = 0.4541, Ka = 0.0178, Ka/Ks = 0.0300; *Saccharomyces cerevisiae *vs. *Pichia stipitis*, Ks = 0.7798, Ka = 0.0221, Ka/Ks = 0.0283). These results suggest that M0880/a and FK01 are significantly diverged from each other and likely constitute distinct species. This hypothesis is consistent with the plastid-derived gene data for rDNA (Fig. 2B) and *fts*Z (Fig. 4). Interestingly, the estimated Ks value between *fts*Z sequences from M0880/a and FK01 exceed the expected confidence limits (i.e., >> 1.0) likely indicating these plastid-encoded genes are undergoing a high nucleotide substitution rate possibly as a result of the genome reduction process. Taken together, our results indicate that after the single acquisition of the photosynthetic organelle, the *P. chromatophora *ancestor gave rise to at least two distinct lineages.

**Figure 5:**
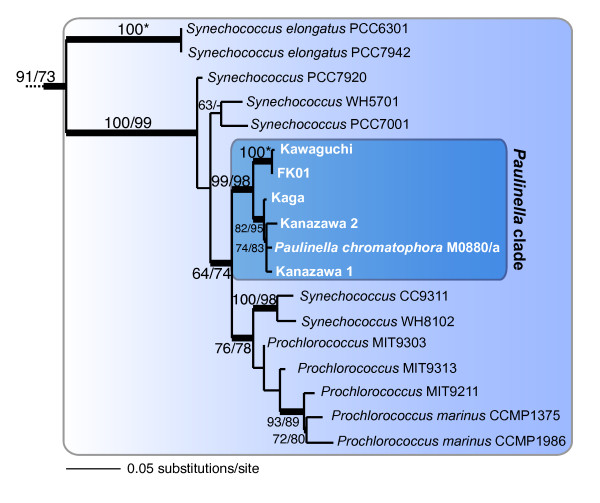
**RAxML phylogenetic tree of plastid 16S rDNA from photosynthetic *Paulinella *spp. with the larger tree (available upon request from HSY) removed at the branch leading to cyanobacteria**. The numbers at the nodes show support values derived from a RAxML bootstrap analysis followed by those from a PhyML analysis. When both values are the same, then this is marked with an asterisk and when a node is not resolved with a method than this is denoted with dashes. Only bootstrap values ≥ 50% are shown. The thick branches have a Bayesian posterior probability > 0.95. Branch lengths are proportional to the number of substitutions per site (see scale bar).

To advance our understanding of photosynthetic *Paulinella*, we generated plastid-encoded 16S rDNA sequences from environmental samples from four different sites in Japan. We amplified the gene directly using a small number of cells that were manually isolated from materials collected at each site (see Methods and Materials). The expanded rDNA tree of photosynthetic *Paulinella *(Fig. 5) shows that all of the isolates cluster together with robust bootstrap and Bayesian support (99% RML, 98% PML) with a sister-group relationship to *Synechococcus/Prochlorococcus*, confirming the single origin of the plastid in these *Paulinella*. The phylogeny also indicates that the *Paulinella *isolates are split into two distinct clades. One clade includes the M0880/a strain that is closely related to isolates from Kaga and Kanazawa-1 and -2, whereas the second includes the newly isolated FK01strain and a field isolate from Lake Kawaguchi (HSY et al. unpublished data). This topology is surprising because it does not provide evidence for geographic separation (i.e., Germany vs. Japan), but rather shows the German strain M0880/a might share a most recent common ancestor with Japanese isolates (i.e., from Kanazawa and Kaga). It is possible, however, that photosynthetic *Paulinella *species are globally distributed and we simply lack data from other sites to demonstrate this result. In any case, our results suggest these fascinating organisms are unlikely to be a relict branch of filose amoebal evolution but may be broadly distributed with many more living taxa than previously thought. Assessing further the biodiversity of this group and providing a taxonomic description of species are key next steps in understanding the biology of *Paulinella *(HSY et al., work underway).

## Conclusion

Our results provide sufficient data to address the major aims of this study. The morphological and molecular data show that M0880/a and the newly isolated FK01 are distinct species (see Table 1), whereas phylogenetic analyses of these strains and other cells collected in nature demonstrate that all of the photosynthetic *Paulinella *share a plastid derived from a single primary endosymbiosis. This is an important finding because the genus *Paulinella *provides a set of diverged lineages that can be used to study the process of post-endosymbiotic plastid evolution using genomic and proteomic methods. One keystone process in this regard is endosymbiotic gene transfer (EGT), driven by a gene transfer ratchet (e.g., [38,39]) that relocates endosymbiont genes to the host nucleus. This is followed in some cases by endosymbiont gene activation and import of the encoded proteins into the plastid. How this occurs in related lineages and which genes are the primary targets for EGT can now be explored in detail by studying the nuclear genome of different photosynthetic *Paulinella*. Nowack and colleagues [22] reported that many cyanobacterial transporters were lost in the endosymbiont genome but the question remains whether they vanished or have been transferred to the nucleus for co-option in other host functions or retargeting to the organelle. A recent study by Nakayama and Ishida [40] described for the first time a nuclear encoded photosynthetic gene in FK01 of cyanobacterial origin. The cDNA encoding psaE contains a putative polyadenylation signal (AGTAAA) and a poly(A) sequence at the 3' terminus, whereas the nuclear locus contains two putative spliceosomal introns. These data provide strong evidence for EGT of the *psa*E gene and we expect to find many more examples of this phenomenon when the nuclear genome of *Paulinella *species is completed (HSY, DB work underway). In this regard, the current tree provides at least two distinct clades in which such questions about post-endosymbiotic EGT can be studied using genomic methods. Preliminary sequence data from the FK01 plastid genome, for example, show that it differs both with respect to gene content and gene order when compared to the published [22] M0880/a genome (ARP, DB, HSY unpublished data).

## Methods

### Sampling and Establishment of the FK01 Strain

*Paulinella chromatophora *strain (FK01) was collected from Daigo, Ibaraki Prefecture, Japan, and cells were isolated into culture by Takeshi Nakayama at the University of Tsukuba. The FK01 strain was then transferred to the Provasoli-Guillard National Center for Culture of Marine Phytoplankton (CCMP). Michael Melkonian from the University of Cologne, Germany kindly provided the German strain M0880/a to the CCMP. Both strains were maintained at the CCMP using DY-V medium at 20°C in flat plastic culture flasks with a 14/10 hr light/dark cycle.

### Light Microscopy

General cell morphology, including the pseudopodia, was examined using a Zeiss Axioimager M1 light microscope.

### Scanning Electron Microscopy

Cells were harvested with centrifugation, fixed (4% osmium tetroxide, 8% glutaraldehyde in TE buffer) and dehydrated (35, 50, 75, 95, and 100% EtOH), mounted on stubs and carbon coated a Denton Vacuum sputter-coater/evaporator (Desk IV, with Carbon Accessory). Cells were observed in a Zeiss Supra 25 field emission SEM.

### DNA Amplification and Sequencing

Total genomic DNA from *Paulinella *cultures was extracted using the DNeasy Plant Mini Kit (Qiagen). PCR was done with degenerate primers for actin (Ac245F: AACTGGGAYGAYATGGARAAGAT; AC1500R: AYCCACATCTGCTGRAANGTG), 18S rDNA (EukA: AACCTGGTTGATCCTGCCAG; EukB: TGATCCTTCTGCAGGTTCACCTAC, [38]) FtsZ (ftsZ150F: AGYAATGCNGTSAAYCGVATGAT; ftsZ1000R: CACGTSACBGTGATYGCCACVGG), and specific primers for the *Paulinella *plastid genes 16S rDNA (16Snt5F: CTTAACACATGCAAGTGCAACG; 16Snt725F: CCATAACTGACGCTCATGGACG; 16Snt725F: CGTCCATGAGCGTCAGTTATGG; 16Snt1500R: GTACGGCTACCTTGTTACGAC) and 23S rDNA (23Snt27F: GATACCTTGGCACACAGAGG; 23Snt1224F: GCAGCTTCGGTAAAACGCTTAG) genes. We amplified and determined partial actin sequences from both *Paulinella *FK01 and M0880/a strains, and we determined partial sequences for the 18S rDNA, 16S rDNA, 23S rDNA, and *fts*Z genes from the FK01 strain. All reactions were done with an initial denaturation step at 94°C for 10 min, followed by 35 cycles of 94°C for 1 min, 50°C for 1 min, and 72°C for 2 min, concluding with a 10 min extension at 72°C. *P. chromatophora *plastid-encoded 16S rDNA sequences were amplified using PCR from four additional isolates from different sites in Japan. The cells were collected from two ponds located in Kanazawa (Kanazawa-1 and -2), from a pond located in Kaga Ishikawa Prefecture, and from Lake Kawaguchi in Yamanashi Prefecture, Japan. Because *P. chromatophora *is difficult to culture in the laboratory, we amplified 16S rDNA fragments directly from isolated cells. To avoid contamination, isolated cells were washed 10 times with sterilized water before mechanical disruption of the scaly cover and the cell membrane. Cell breakage was corroborated by light microscopy. The cell homogenate was transferred directly to microfuge tubes to be used as DNA template for PCR amplification using universal primers (U16F1: AGAGTTTGATCCTGGCTCAG, U16R1: ACGGCTACCTTGTTACGACTT). PCR products were purified (QIAquick PCR Purification kit, Qiagen) and either directly sequenced (BigDye™ Terminator Cycle Sequencing Kit, PE-Applied Biosystems) or cloned (TOPO 4 PCR vector, Invitrogen) prior to sequencing. All sequences are available in GenBank (accession numbers from FJ456915 – FJ456920 and FJ184058 – FJ184061).

### Phylogenetic Analysis

Sequence sets (protein or nucleotide) were aligned with Muscle [41], and manually refined (alignments are available upon request). The ML trees were inferred with RAxML (VI-HPC, v2.2.1, [42]) using the WAG substitution model, gamma distribution ('PROTGAMMAWAG' implementation), with 4 discrete rate categories, and starting from a random tree. Branch support was evaluated with 100 bootstrap replicates using both RAxML (WAG substitution model and the 'PROTCATWAG' implementation) and PhyML [43] (WAG + Γ substitution model, and parameters estimated during the tree search). We also did Bayesian analyses with each data set using MrBayes 3.1.1 [44] and the WAG + I + Γ model of sequence evolution for the protein alignments and GTR I + Γ for the rDNA data. For each alignment, Metropolis-coupled Markov chain Monte Carlo from a random starting tree and 2 runs were started simultaneously. The Bayesian analyses were run for 1,000,000 generations with trees sampled each 100 cycles. Four chains were run simultaneously of which three were heated and one was cold, with the initial 250,000 cycles (2,500 trees) being discarded as the 'burn in'. A consensus tree was made with the remaining phylogenies to determine the posterior probabilities at each node.

### Nucleotide Substitution Rate Calculation

Actin sequences at both protein and nucleotide levels of *Ostreococcus tauri *(jgi, Ostta4 29599), *Ostreococcus lucimarinus *(gi, 144581780), *Saccharomyces cerevisiae *(gi, 42742172), *Pichia stipitis *(gi, 126257874), and *Kluiveromyces lactis *(gi, 50306650) were obtained from JGI and GenBank. Paired protein sequences were aligned with Muscle [41], thereafter, protein pairwise alignments were used as a reference to align the corresponding coding nucleotide sequences to identify the correct reading frame. The rate of synonymous (Ks) and non-synonymous (Ka) substitutions were estimated from the resulting nucleotide alignments using PAML [45].

## Authors' contributions

HSY oversaw data collection and analysis, led the morphological analysis, contributed to writing; ARP led the molecular phylogenetic analysis, contributed to writing; RAA contributed to culture maintenance and SEM analysis, and contributed to writing; SMB contributed to financial support for the *Paulinella *gene sequencing, and contributed to writing; KI contributed to culture establishment of FK01; TN contributed to culture establishment of FK01 and generated 16S rDNA sequences from four additional isolates; DB oversaw data collection and analysis, contributed to writing and revision.
